# Predicting in-hospital mortality for sepsis: a comparison between qSOFA and modified qSOFA in a 2-year single-centre retrospective analysis

**DOI:** 10.1007/s10096-020-04086-1

**Published:** 2020-10-28

**Authors:** Matteo Guarino, Edoardo Gambuti, Franco Alfano, Alfredo De Giorgi, Elisa Maietti, Andrea Strada, Francesco Ursini, Stefano Volpato, Giacomo Caio, Carlo Contini, Roberto De Giorgio

**Affiliations:** 1 Department of Morphology, Surgery and Experimental Medicine, St. Anna University Hospital, University of Ferrara, Via A. Moro, 844124, Cona, Ferrara, Italy; 2 Department of Internal Medicine, St. Anna University Hospital, University of Ferrara, Cona, Ferrara, Italy; 3 Department of Biomedical and Neuromotor Sciences, University of Bologna, Bologna, Italy; 4 Centre of Clinical Epidemiology, Department of Medical Science, University of Ferrara, Ferrara, Italy; 5 Department of Emergency Medicine, St. Anna University Hospital, University of Ferrara, Cona, Ferrara, Italy; 6 Department of Infectious and Dermatology Diseases, St. Anna University Hospital, University of Ferrara, Cona, Ferrara, Italy

**Keywords:** Sepsis, Septic shock, SpO2/FiO2, In-hospital mortality, Patients’ outcome, MqSOFA

## Abstract

Sepsis is a life-threating organ dysfunction caused by a dysregulated host response to infection. This study proposed a new tool, i.e. modified qSOFA, for the early prognostic assessment of septic patients. All cases of sepsis/septic shock consecutively observed in 2 years (January 2017–December 2018), at St. Anna University Hospital of Ferrara, Italy, were included. Each patient was evaluated with qSOFA and a modified qSOFA (MqSOFA), i.e. adding a SpO2/FiO2 ratio to qSOFA. Logistic regression and survival analyses were applied to compare the two scores. A total number of 1137 consecutive cases of sepsis and septic shock were considered. Among them 136 were excluded for incomplete report of vital parameters. A total number of 668 patients (66.7%) were discharged, whereas 333 (33.3%) died because of sepsis-related complications. Data analysis showed that MqSOFA (AUC 0.805, 95% C.I. 0.776–0.833) had a greater ability to detect in-hospital mortality than qSOFA (AUC 0.712, 95% C.I. 0.678–0.746) (*p* < 0.001). Eighty-five patients (8.5%) were reclassified as high-risk (qSOFA< 2 and MqSOFA≥ 2) resulting in an improvement of sensitivity with a minor reduction in specificity. A significant difference of in-hospital mortality was observed between low-risk and reclassified high-risk (*p* < 0.001) and low-risk *vs.* high-risk groups (*p* < 0.001). We demonstrated that MqSOFA provided a better predictive score than qSOFA regarding patient’s outcome. Since sepsis is an underhanded and time-dependent disease, physicians may rely upon the herein proposed simple score, i.e. MqSOFA, to establish patients’ severity and outcome.

**Supplementary Information:**

The online version contains supplementary material available at 10.1007/s10096-020-04086-1.

## Introduction

Sepsis is a life-threating organ dysfunction resulting from a dysregulated host response to a wide range of infections. It is a major public health concern, accounting for more than $20 billion (5.2%) of the total US hospital costs in 2011 [1, 2]. Sepsis has an incidence of 4 per 1000 individuals in the Italian population [3] with a mortality rate increasing from 18.7 to 49.3% in the last 15 years (ISTAT 2019). Despite these alarming data, nearly 90% of the general population has no clue about the word “sepsis” and 58% did not consider sepsis a cause of death [4]. Likewise, only 17% of physicians agreed upon any currently available definition of sepsis [5]. In the last 30 years, the definition of sepsis changed as well as its diagnostic criteria. After the third international consensus on sepsis and septic shock (Sepsis-3) [1], experts reached a consensus on a quick sequential organ failure assessment (qSOFA), i.e. a useful score to approach patients admitted in non-intensive care unit (ICU) setting whose infection is thought to evolve into sepsis. Since a qSOFA ≥2 alerts on the high probability of sepsis in a given patient (increasing the risk of mortality of approximately 10%), physicians should determine the sequential organ failure assessment (SOFA) score in order to establish the overall organ dysfunction. A clinical picture of sepsis is established when SOFA score is ≥ 2, whereas septic shock is defined by a vasopressor (e.g. norepinephrine) maintained mean arterial pressure (MAP) ≥65 mmHg and serum lactate level ≥ 2 mmol/L [1, 6]. Comparing the diagnostic criteria of Sepsis-3 with those of Sepsis-2, qSOFA and SOFA showed a better identification of septic patients than previous criteria, such as systemic inflammatory response symptoms (SIRS) [7]. However, from 2017, some authors raised concern about the prognostic value of the qSOFA and SOFA in terms of mortality [8–15]. In the ICU setting, a SOFA score ≥ 2 demonstrated a greater prognostic accuracy for in-hospital mortality than SIRS and qSOFA, thus dampening the value of the latter, which is commonly used in clinical practice [8]. Moreover, in one study, qSOFA failed to identify two thirds of patients with severe sepsis admitted to emergency units [9], and a qSOFA score ≥ 2 exhibited poor sensitivity in detecting septic patients in either pre-hospital or emergency setting, thus providing further evidence that Sepsis-2 and Sepsis-3 criteria are comparable in predicting mortality [10–12]. Compared with qSOFA criteria, the advantage of the SIRS criteria is determined by a greater clinical information on the number and type of organ dysfunction [13]. Other authors tried to compare qSOFA to other existing scores such as the NEWS [14] and the GYM [15], showing that NEWS did not differ from qSOFA in establishing hospitalization and mortality of septic patients, whereas qSOFA demonstrated a lower sensitivity than GYM despite a better specificity [14, 15]. Another study based on a retrospective analysis of 1865 septic patients scored by qSOFA, SOFA, lactate and lactate added to qSOFA showed that only the lactate levels had a superior prognostic accuracy for short-term and long-term mortality than any other criteria, including qSOFA [16]. The combination of lactate assay with qSOFA showed a predictive in-hospital mortality close to that of the SOFA score alone. Based on this background and because of the need of an easy tool useful to detect patients with suspected infection and high risk of in-hospital mortality, we designed the present study aimed to propose a modified qSOFA (MqSOFA). In addition to classic items, i.e. blood pressure, respiratory rate and change in mental status, we included the SpO2/FiO2 (S/F) ratio, which focuses on peripheral oxygen saturation and fraction of inhaled oxygen. The S/F ratio is known to be a good predictive factor of respiratory dysfunction in most patients [17]. According to Sepsis-3, qSOFA is recommended in daily emergency practice to establish the severity of patients with sepsis [1, 6]; thus our primary goal was to compare MqSOFA *vs.* qSOFA score in predicting the overall risk of in-hospital mortality. The secondary aim was to assess the length of stay (LOS) of patients by comparing MqSOFA *vs.* qSOFA. To achieve the main goal of this study, the investigated cohort was stratified into two subgroups as follows, i.e. the first with a qSOFA or MqSOFA < 2, the second with ≥ 2, in order to demonstrate how this cut-off can be useful to identify populations at greater risk of in-hospital mortality and/or increased length of stay.

## Material and methods

This is a retrospective observational study. All patients with sepsis or septic shock (according to the International Classification of Diseases, 9th Revision, Clinical Modification, ICD9-CM code: 995.91 and 785.52, respectively) diagnosed from January 2017 to December 2018 and admitted to the Emergency Department of St. Anna Hospital, Cona, Ferrara, Italy, were included for this single-centre study. Ferrara is small town located in the Emilia-Romagna Region (Northern Italy) with an almost exclusively Caucasian population of approximately 150,000 inhabitants. St. Anna is the University Hospital, the main medical community institution in the province and the major referral centre, which serves a global population of over 350,000 people. Every year the Emergency Department of the St. Anna Hospital admits about 50,000 patients of whom 33% are > 75 years of age. A considerable number of these patients (nearly 40% of the total admissions) are classified as ‘yellow code’ (i.e. an intermediate emergency class code) with a mean time of 50 min for the first medical evaluation. Cases of sepsis occurred during the period of study were identified by searching for diagnosis of ‘sepsis’ and ‘septic shock’ on the discharge letter from the emergency department. Using such methodology, we retrieved a total of 1137 individual records; of this, 1001 had full information available to retrospectively calculate qSOFA and MqSOFA. Intubated patients were not recruited in this study. Each patient included in this study was evaluated with qSOFA and MqSOFA (the main differences between these two scores are summarized in Table 1). In the MqSOFA computation, a score of 0 is added if the S/F ratio is ≥ 315, 1 point if it is between 314 and 236 and 2 points if it is ≤ 235. Previous data indicated that S/F ratios of 235 and 315 correlate with PaO2/FiO2 (P/F) ratios of 200 and 300 in patients with acute respiratory distress syndrome (ARDS) or lung injury. The S/F ratio is non-invasive, rapidly assessed (via pulse oximetry) and repeatable index to evaluate the respiratory function [17].

**Table 1:** Comparison between the parameters taken into consideration by qSOFA and MqSOFA

qSOFA		MqSOFA		
Parameter	Points	Parameter		Points
Blood pressure ≤ 100 mmHg	1	Blood pressure ≤ 100 mmHg		1
Respiratory rate ≥ 22/min	1	Respiratory rate ≥ 22/min		1
Altered mentation	1	Altered mentation		1
		SpO2/FiO2 ratio	≥ 316	0
			236–315	1
			≤ 235	2

## Statistical analysis

Categorical data were expressed as absolute frequencies and percentages, while means ± SD or median and inter-quartile range (IQR) were reported for continuous variables, as appropriate. To denote differences between survivors and patients died because of sepsis severity and related complications, the two groups were compared with respect to sex and age using the Pearson’s *X*^2^ and Mann-Whitney tests as appropriate. The association between in-hospital mortality and the two qSOFA scores was investigated with univariated and multivariated logistic regression analysis. Odds ratios (ORs) and their 95% confidence intervals were reported. Moreover, the AUC of the ROC curves were compared in order to identify the tool with the best discriminative ability. Patients were then grouped as follows: ‘low-risk’ (qSOFA< 2 and MqSOFA< 2), ‘reclassified high-risk’ (qSOFA< 2 and MqSOFA≥ 2) and ‘high-risk’ (qSOFA≥ 2 and MqSOFA≥ 2). Survival probability in these three groups was compared using Kaplan-Meier curves and the log rank test. The association with length of stay (LOS) was analysed only for survivors. The median LOS and related IQR was reported and compared between groups using the Wilcoxon-Mann-Whitney test (*k* = 2 groups) and the Kruskal-Wallis test (*k* > 2 groups) as appropriate. Sensitivity analyses on two subgroups, young patients (< 70 years old) and very old patients (≥ 90 years) were conducted in order to check for results consistency. Stata 13.0 (StataCorp LLC, College Station, TX) was used for statistical analyses and the significance level was set for *p* < 0.05.

## Results

**Fig. 1:**
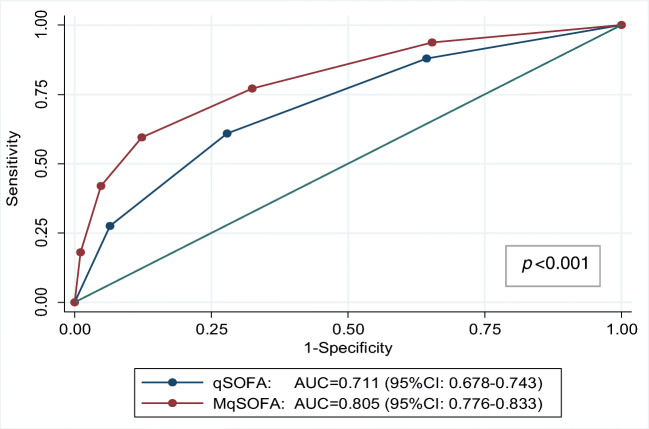
Comparison of ROC curves of qSOFA and MqSOFA to assess in-hospital mortality

Among the 1001 patients analysed, 462 were males (46.2%) and 539 females (53.8%) with a mean age of 79.4 ± 12.9 years (range 19–99 years). A total number of 668 patients (66.7%) were discharged, whereas 333 (33.3%) died because of sepsis-related complications (mostly multi-organ failure). There were no statistically significant differences in terms of mortality between male and female gender (*p* = 0.242). In contrast, in the subset with fatal outcome, age was significantly higher *vs.* those who were discharged (82.4 ± 10.7 years *vs.* 77.9 ± 13.6 years; *p* < 0.001), resulting a discriminant factor that negatively influenced the outcome (OR 1.03, 95% C.I. 1.02–1.04; *p* < 0.001). The distribution of patients according to qSOFA and MqSOFA has been reported in Table 2. Considering sepsis-diagnostic scores as predictors of in-hospital mortality, the OR for one-unit increase in the score was 2.29 (95% C.I. 1.97–2.65, *p* < 0.001) for qSOFA and 2.38 (95% C.I. 2.12–2.67, *p* < 0.001) for MqSOFA; results were consistent after the adjustment for age (Table 3). The Hosmer-Lemeshow test was not significant for both scores, thus indicating a good calibration. The area under the curve (AUC) shown in Fig. 1, the MqSOFA score (AUC 0.805, 95% C.I 0.776–0.833) had a greater ability to detect in-hospital mortality than qSOFA (AUC 0.712, 95% C.I 0.678–0.746) (*p* < 0.001). Comparing the ‘low-risk’ and ‘high-risk’ groups, both qSOFA and MqSOFA scores ≥ 2 represented a robust negative predictive factor (OR 4.0, 95% C.I: 3.1–5.3 *p* < 0.001 *vs.* OR 7.0, 95% C.I: 5.2–9.5, *p* < 0.001) for in-hospital mortality, even after the adjustment for age. Indeed, a qSOFA ≥ 2 showed a sensitivity and specificity of 61 and 72%, respectively (accuracy 68.4%, positive predictive value 52.1%, negative predictive value 78.8%); whereas sensitivity and specificity with MqSOFA for the same cut-off were 77.2 and 67.5%, respectively (accuracy 70.7%, positive predictive value 54.2%, negative predictive value 85.6%). MqSOFA ≥ 2 resulted in a sensitivity gain of 16% and a loss of 5% in specificity (Table 4). Eighty-five patients (8.5%) were reclassified as high risk resulting in an improvement of accuracy and sensitivity with a minor reduction in specificity.

**Table 2:** Patients distribution according to qSOFA and MqSOFA

	qSOFA				Total
MqSOFA	0	1	2	3	
0	252	0	0	0	252
1	11	264	0	0	275
2	15	43	136	0	194
3	0	27	50	31	108
4	0	0	68	37	105
5	0	0	0	67	67
**Total**	278	334	254	135	1001

**Table 3:** Logistic regression analysis of in-hospital mortality

	Univariate model			Multivariate/age-adjusted model		
	OR	95%C.I.	*p*	OR	95%C.I.	*p*
**qSOFA**	2.29	(1.97–2.65)	< 0.001	2.20	1.90–2.56	< 0.001
**MqSOFA**	2.38	(2.12–2.67)	< 0.001	2.34	2.08–2.63	< 0.001

**Table 4:** Sensitivity, specificity and accuracy of qSOFA ≥ 2 and MqSOFA ≥ 2

	Sensitivity	Specificity	Accuracy	PPV	NPV
**qSOFA**	61.0%	72.2%	68.4%	52.1%	78.8%
**MqSOFA**	77.2%	67.5%	70.7%	54.2%	85.6%

*PPV* positive predictive value, *NPV* negative predictive value

*p* < 0.001

**Fig. 2:**
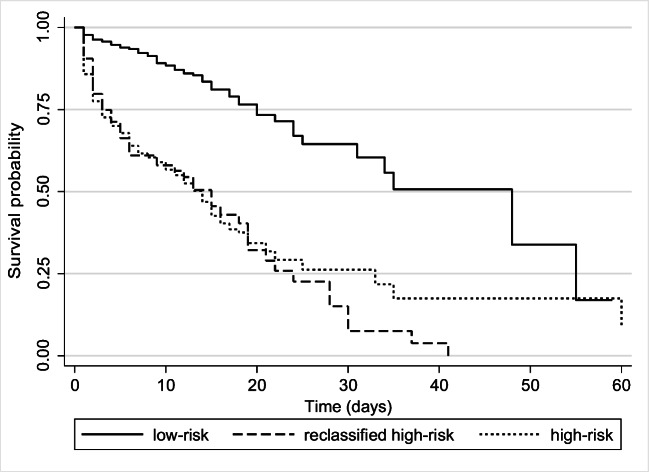
Secondary survival analysis. Stratification of the cohort into low-risk (qSOFA < 2 and MqSOFA < 2), reclassified high-risk risk (qSOFA < 2 and MqSOFA ≥ 2) and high-risk groups (qSOFA ≥ 2 and MqSOFA ≥ 2)

Figure 2 illustrates three survival curves (Kaplan-Meier) generated after the stratification of the cohort into low-risk, reclassified high-risk risk and high-risk groups. At the log-rank test, we found no differences in terms of in-hospital mortality between high-risk and reclassified high-risk groups (*p* = 0.551) and a significant difference in survival between low-risk and reclassified high-risk (*p* < 0.001) as well as between low-risk and high-risk groups (*p* < 0.001). The median LOS for discharged patients was 8.5 days [IQR: 6-13] for low risk, 10 days [IQR: 6–13] for reclassified high risk and 10 days for high risk [IQR: 7–14] group (*p* = 0.009).

Sensitivity analysis has been reported in the Supplementary File. Results among young patients (< 70 years old) and very old patients (≥ 90 years) did not show any statistical differences when compared with the whole cohort included in this study.

## Discussion

Sepsis is often an underestimated condition because of its insidious clinical picture and should always be suspected in patients with clear signs of infection to establish appropriate management beginning from the emergency setting [1–3]. Despite Sepsis-3, which provided the basis for SOFA and qSOFA scores, the early diagnosis of sepsis remains a challenge for physicians. Furthermore, the prognostic value in terms of mortality of qSOFA and SOFA has been questioned [8–16]. Despite some authors provided evidence hampering the role of qSOFA and SOFA, others continued to show the prognostic validity of these scores [17–25]. The great advantage of qSOFA score relies upon its simple structure (few items), low cost (clinical features) and easy calculation without the need of blood tests [19, 20]. A recent meta-analysis indicated that qSOFA ≥ 2 and SIRS ≥ 2 were associated with an increased mortality in patients with infectious disease. The high specificity of the qSOFA may help physicians to identify patients who require accurate clinical monitoring. Nonetheless, because of the high mortality rate of patients with sepsis, a sensitive test/score should be available to physicians, particularly those working in the emergency setting [26]. Based on the meta-analysis by Freund et al. [18], the present study suggests a modification of qSOFA by adding the SpO2/FiO2 (or more simply ‘S/F ratio’) to the previous criteria. The S/F parameter is not an innovation and it was proposed by some authors as a non-invasive, repeatable and rapid criterion to assess the respiratory function in different diseases, such as ARDS or venous thromboembolism [17, 27–33]. The best fitting association between S/F and PaO2/FiO2 (or ‘P/F’, usually applied in clinical practice to assess respiratory function) ratios was described by a linear relationship between the transformed logarithmic value of S/F and P/F ratios, with the regression equation Log(P/F) = 0.48 + 0.78 × Log(S/F) and R-square of 0.31 [33]. As already mentioned, Rice et al. showed that S/F ratios of 235 and 315 correlate with P/F ratios of 200 and 300 in patients with ARDS or lung injury [17]. There is a wide range of variation in the relationship between S/F and P/F ratios in patients with non-respiratory deficits [17]. Other possible limitations of the SpO2/FiO2 ratio concern the use of pulse oximeter. Indeed, a falsely normal SpO2 value may result from an incorrect oximetry occurring in various conditions/diseases, e.g. carbon monoxide poisoning or sickle cell anaemia. SpO2 value may also be falsely low in paradoxical pulse (also referred to as ‘venous pulsation’), severe anaemia with concomitant hypoxia or even poor fingernail cleaning. Finally, other conditions that may alter SpO2 value are methemoglobinemia, sulfhemoglobinemia, severe hyperbilirubinemia, circulating foetal haemoglobin as well as septic shock and other causes of poor perfusion [33]. The present study confirmed that patients with a high qSOFA score (≥ 2) showed an increased mortality as indicated by Singer et al. in Sepsis-3 [1]. However, compared with qSOFA our data revealed that MqSOFA provided a better measure of in-hospital mortality risk. The introduction of the S/F ratio to qSOFA added an important parameter to better assess respiratory dysfunction that is related to worsening of the clinical outcome, thus providing greater sensibility than qSOFA.

MqSOFA showed an increased sensitivity and a lower specificity than qSOFA. The ‘ideal MqSOFA’ should lead to an improvement in sensitivity without lowering the specificity. In this work we developed the MqSOFA with the intent to minimize the possible underestimation of high-risk mortality cases; thus a small reduction in specificity with a relevant improvement in sensitivity can be tolerable. Indeed, the data of the present study showed a gain in sensitivity of about 16% with a loss in specificity of about 5% (as reported in Table 4). Furthermore, MqSOFA provided valuable data on in-hospital length of stay, which was the secondary outcome of our study. We showed that the length of stay progressively increased in parallel with MqSOFA scores. In particular, in the three stratified patient subgroups, we showed a statistically significant difference in terms of LOS indicating that a high value of MqSOFA can accurately identify a longer in-hospital LOS for septic patients. In our cohort, in-hospital mortality was not related to gender, whereas age resulted to be a factor that impacted negatively the septic patient outcome. This result is in line with the evidence that elderly patients are usually burdened by a large number of comorbidities (e.g. chronic renal failure or coronary artery disease), which can worsen in-hospital mortality [34, 35].

Finally, showing that a subset of patients with an apparently uneventful qSOFA may have a poor outcome when re-classified with MqSOFA provided further strength to our study. Specifically, our data supported that MqSOFA can more accurately stratify patients at high-risk of in-hospital mortality compared with qSOFA. Using MqSOFA may allow physicians to guarantee a better management to patients with sepsis, hence improving their outcome.

## Conclusion

In this single-centre study performed in the Northern East area of the Emilia-Romagna region of Italy, we confirmed that, compared with qSOFA, MqSOFA (and in particular if the score ≥ 2) provides a better definition of patient outcome in terms of in-hospital mortality. Since sepsis is often misrecognized and a timely established assessment of the risk of septicaemia is mandatory in the emergency setting, we proposed a simple and inexpensive score, i.e. the MqSOFA, to better determine a possible unfavourable outcome. Also, our data showed that reclassified high-risk and high-risk subgroups had longer LOS compared with low-risk patients. Clearly our study has limitations: it is a retrospective analysis and a single-centre database, which considerably reduced the statistical power of this investigation. Also, the S/F ratio has limitations regarding the SpO2 parameter and its high variability in relation to different clinical conditions. Nonetheless, we consider S/F ratio useful for its simple assessment, making MqSOFA a possibly valuable tool for physicians.

Future prospective studies, performed on large cohort, are eagerly awaited to prove the actual efficacy of MqSOFA in predicting the outcome of patients with sepsis.

## Electronic supplementary material

Supplementary file Sensitivity analysis among patients under 70 years (sensitivity analysis 1) and over 90 years (sensitivity analysis 2). (DOCX 34 kb)

## Data Availability

The datasets generated and/or analysed during the current study are not publicly available due to privacy policy but are available from the corresponding author on reasonable request.

## List of abbreviations

ARDS: Acute respiratory distress syndrome
AUC: Area under the curve
ED: Emergency department
FiO2: Inspired fraction of oxygen
FO2Hb: Fraction of oxygen bind to haemoglobin
GYM: Glasgow; tachYpnea; Morbidity
ICU: Intensive care unit
IQR: inter-quartile range
LOS: Length of stay
MAP: Mean arterial pressure
MqSOFA: Modified quick sequential organ failure assessment
NEWS: National early warning score
OR: Odds ratio
qSOFA: Quick sequential organ failure assessment
ROC: Receiver operating characteristic
SIRS: Systemic inflammatory response syndrome
SOFA: Sequential organ failure assessment
SpO2: Peripheral oxygen saturation
PaO2: Arterial partial pressure of oxygen
