# Supplementary material for: Predicting in-hospital mortality for sepsis: a comparison between qSOFA and modified qSOFA in a 2-year single-centre retrospective analysis
Source: Eur J Clin Microbiol Infect Dis. 2020 Oct 28;40(4):825–31. doi: 10.1007/s10096-020-04086-1 (PMC7979592; doi:10.1007/s10096-020-04086-1)
Supplement: Supplementary file 1 — Sensitivity analysis among patients under 70 years (sensitivity analysis 1) and over 90 years (sensitivity analysis 2). (DOCX 34 kb) [file 10096_2020_4086_MOESM1_ESM.docx]

**Supplementary file.** Sensitivity analysis among patients under 70 years (sensitivity analysis 1) and over 90 years (sensitivity analysis 2).

**Sensitivity analysis 1**

Comparison between qSOFA and MqSOFA among the subgroups of 169 patients <70 years.

**Table S1.** Logistic regression analysis of in-hospital mortality in patients <70 years. OR for one-unit increase in the score.

|  | **Univariate model** | | | **Multivariate/age-adjusted model** | | |
| --- | --- | --- | --- | --- | --- | --- |
|  | **OR** | **95%C.I.** | ***p*** | **OR** | **95%C.I.** | ***p*** |
| **qSOFA** | 2.56 | 1.70 – 3.85 | <0.001 | 2.58 | 1.71 – 3.89 | <0.001 |
| **MqSOFA** | 2.42 | 1.78 – 3.29 | <0.001 | 2.41 | 1.78 – 3.28 | <0.001 |

**Figure S1.** Comparison of ROC curves of qSOFA and MqSOFA to assess in-hospital mortality (p=0.002), in patients <70 years.

**Table S2.** Distribution of patients according to qSOFA and MqSOFA < or ≥2, in patients <70 years.

|  | qSOFA<2 | qSOFA≥2 |
| --- | --- | --- |
| MqSOFA<2 | 116 | 0 |
| MqSOFA≥2 | 10 | 43 |

**Table S3.** Sensitivity, specificity and accuracy of qSOFA≥2 and MqSOFA≥2, in patients <70 years.

|  | **Sensitivity** | **Specificity** | **Accuracy** | **PPV** | **NPV** |
| --- | --- | --- | --- | --- | --- |
| **qSOFA** | 50.0% | 81.2% | 74.6% | 41.9% | 85.7% |
| **MqSOFA** | 66.7% | 78.2% | 75.7% | 45.3% | 89.7% |

PPV: positive predictive value; NPV: negative predictive value

**Figure S2.** Kaplan-Meier survival curves for in-hospital mortality. Stratification of the cohort of patients <70 years into low-risk (qSOFA<2 and MqSOFA<2), reclassified high-risk (qSOFA<2 and MqSOFA≥2) and high-risk groups (qSOFA≥2 and MqSOFA≥2).

Both scores were significant predictors of in-hospital mortality among patients <70 years (Table S1). MqSOFA resulted in a higher AUC (*p*=0.002, Figure S1). Considering a score ≥2, 10 patients (6%) were reclassified as high-risk (qSOFA<2 and MqSOFA ≥2) (Table S2), resulting in similar accuracy and an improvement of 17% in sensitivity with a minor reduction (-3%) in specificity (Table S3).

Survival curves were reported in Figure S2. There was a significant difference between low-risk and the other two groups (*p*<0.001), while the difference between reclassified high-risk and high-risk groups was not significant (*p*=0.168).

Results in the subgroup of patients <70 years were consistent with overall analysis.

**Sensitivity analysis 2**

Comparison between qSOFA and MqSOFA in the subgroup of 199 patients with ≥90 years.

**Table S4.** Logistic regression analysis of in-hospital mortality patients with ≥90 years. OR for one-unit increase in the score.

|  | **Univariate model** | | | **Multivariate/age-adjusted model** | | |
| --- | --- | --- | --- | --- | --- | --- |
|  | **OR** | **95%C.I.** | ***p*** | **OR** | **95%C.I.** | ***p*** |
| **qSOFA** | 2.40 | 1.70 – 3.40 | <0.001 | 2.41 | 1.70 – 3.41 | <0.001 |
| **MqSOFA** | 2.50 | 1.90 – 3.28 | <0.001 | 2.49 | 1.90 – 3.27 | <0.001 |

**Figure S3.** Comparison of ROC curves of qSOFA and MqSOFA to assess in-hospital mortality (*p*<0.001), in patients with ≥90 years.

**Tabel S5.** Distribution of patients according to qSOFA and MqSOFA < or ≥2, in patients with ≥90 years.

|  | qSOFA<2 | qSOFA≥2 |
| --- | --- | --- |
| MqSOFA<2 | 85 | 0 |
| MqSOFA≥2 | 19 | 95 |

**Table S6.** Sensitivity, specificity and accuracy of qSOFA≥2 and MqSOFA≥2, in patients with ≥90 years.

|  | **Sensitivity** | **Specificity** | **Accuracy** | **PPV** | **NPV** |
| --- | --- | --- | --- | --- | --- |
| **qSOFA** | 69.9% | 68.1% | 68.8% | 61.1% | 76.0% |
| **MqSOFA** | 83.1% | 61.2% | 70.4% | 60.5% | 83.5% |

PPV: positive predictive value; NPV: negative predictive value

**Figure S4.** Kaplan-Meier survival curves for in-hospital mortality. Stratification of the cohort of patients with ≥90 years into low-risk (qSOFA<2 and MqSOFA<2), reclassified high-risk (qSOFA<2 and MqSOFA≥2) and high-risk groups (qSOFA≥2 and MqSOFA≥2).

Both scores were significant predictors of in-hospital mortality in patients with ≥90 years (Table S4). MqSOFA resulted in a higher AUC (*p*<0.001, Figure S3). Considering a score ≥2, 19 patients (10%) were reclassified as high-risk, (qSOFA<2 and MqSOFA ≥2) (Table S5), resulting in similar accuracy and an improvement of 13% in sensitivity with a minor reduction (-7%) in specificity (Table S6).

Survival curves were reported in Figure S4. There was significant difference between low-risk and the other two groups (*p*<0.01), while the difference between reclassified-high-risk and high-risk groups was not significant (*p*=0.477).

Results among the subgroup of patients with ≥90 years were consistent with overall analysis.
